# Cytosolic Guanine Nucledotide Binding Deficient Form of Transglutaminase 2 (R580a) Potentiates Cell Death in Oxygen Glucose Deprivation

**DOI:** 10.1371/journal.pone.0016665

**Published:** 2011-01-31

**Authors:** Gozde Colak, Jeffrey W. Keillor, Gail V. W. Johnson

**Affiliations:** 1 Department of Pharmacology and Physiology, University of Rochester, Rochester, New York, United States of America; 2 Département de Chimie, Université de Montréal, Montréal, Canada; 3 Department of Anesthesiology, University of Rochester, Rochester, New York, United States of America; Texas A&M University, United States of America

## Abstract

Transglutaminase 2 (TG2) is a hypoxia-responsive protein that is a calcium-activated transamidating enzyme, a GTPase and a scaffolding/linker protein. Upon activation TG2 undergoes a large conformational change, which likely affects not only its enzymatic activities but its non-catalytic functions as well. The focus of this study was on the role of transamidating activity, conformation and localization of TG2 in ischemic cell death. Cells expressing a GTP binding deficient form of TG2 (TG2-R580A) with high basal transamidation activity and a more extended conformation showed significantly increased cell death in response to oxygen-glucose deprivation; however, targeting TG2-R580A to the nucleus abrogated its detrimental role in oxygen-glucose deprivation. Treatment of cells expressing wild type TG2, TG2-C277S (a transamidating inactive mutant) and TG2-R580A with Cp4d, a reversible TG2 inhibitor, did not affect cell death in response to oxygen-glucose deprivation. These findings indicate that the pro-cell death effects of TG2 are dependent on its localization to the cytosol and independent of its transamidation activity. Further, the conformational state of TG2 is likely an important determinant in cell survival and the prominent function of TG2 in ischemic cell death is as a scaffold to modulate cellular processes.

## Introduction

Transglutaminase 2 (TG2) is a multifunctional protein which plays a role in many different cellular processes including differentiation, neuronal growth, inflammation, development, wound healing [1] and hypoxic cell response [2]. In addition to catalyzing calcium-dependent transamidation reactions, TG2 binds and hydrolyzes GTP and GTP binding inhibits the transamidation activity [3]. Under normal physiological conditions, due to low calcium levels and high GTP levels, TG2 is a latent enzyme with respect to transamidation activity [4], [5]. Under pathological conditions with high intracellular calcium and decreased GTP reserves, increases in TG2 transamidation activity likely occur [6]. A significant outcome of calcium binding is that concurrent with activation, TG2 undergoes an extraordinary conformational change that results in an extended structure [7]. In contrast, in the GTP bound state, TG2 exists in a compact and closed structure that decreases the accessibility of the active site [8], [9]. Therefore, calcium binding and GTP binding inversely regulate the conformational state of TG2, as well as the transamidation activity.

In addition to its enzymatic activities, TG2 can also act as a scaffold or linker protein to mediate protein-protein interactions both extracellularly [10], [11] and intracellularly [2], [12], [13]. TG2 contributes to the organization of the extracellular matrix via binding to fibronectin and mediating its interaction with collagen and integrins [10], [14], [15]. These interactions primarily play a role in migration and wound healing independent of its transamidation activity or GTP binding ability [16]. In the nucleus, TG2 interacts with c-Jun and this interaction can interfere with c-Jun binding to AP-1 binding sites on promoters. This leads to decreased matrix metalloproteinase-9 (MMP-9) expression [13]. TG2 co-immunoprecipitates with Rb protein, and E2F transcriptional activity is significantly suppressed in cells expressing nuclear localized wild-type TG2 [12]. Recently it was shown that wild type TG2 suppresses cytochrome c promoter reporter activity when mutant huntingtin is present [17]. Further, TG2 interacts with HIF-1β, the constitutively expressed subunit of HIF-1 (Hypoxia inducible factor-1) transcription factor, and attenuates hypoxic signaling in SH-SY5Y cells independent of its transamidating activity [2]. In addition there are HREs in the TG2 promoter [18] and TG2 is upregulated in stroke models [19], [20], [21], [22]. These findings indicate that TG2 is a hypoxia responsive protein that may modulate transcriptional activity of hypoxia responsive genes via its interaction with HIF-1β.

Previously it was shown that nuclear localization of TG2 can play a role in cell survival in a transamidation inactive state [12]. Increased nuclear localization of TG2 was detected in SH-SY5Y cells in response to hypoxia concurrent with protection against oxygen-glucose deprivation (OGD)-induced cell death [2]. In a mouse model, nuclear translocation of exogenously expressed human TG2 was observed after middle cerebral artery ligation (MCAL) concomitant with protection against stroke damage [20]. These findings suggest that the cellular localization of TG2 may be important in determining whether TG2 will facilitate or ameliorate cell death processes, particularly in response to OGD.

In previous studies, it was shown that R580A mutation of human TG2 (TG2-R580A) prevents GTP binding. Rat TG2 with this same mutation (R579A) exhibited higher transamidation activity at basal conditions compared to wild type TG2 [23]. Additionally, R579A exhibited an open conformation due to absence of GTP binding which is the stabilizing factor for the closed structure [9]. Therefore, forms of TG2 that are deficient in GTP binding can show high basal transamidation activity. In another study, it was found that R580L and R580K mutants of human TG2 cause increased cell death in response to serum deprivation [24]. These data suggest that the absence of GTP binding can potentiate cell death under stress conditions. However, it is still not known whether the detrimental affect of TG2-R580A is due to its high transamidation activity, lack of GTP binding ability or its open conformational state.

In this study, we investigated the role of intracellular localization, transamidation activity and different conformations of TG2 in its protective effect in OGD induced cell death. In a clonal striatal cell model, TG2 was not translocated into the nucleus in response to OGD and neither wild type TG2 nor a transamidating inactive form (TG2-C277S) protected against OGD induced cell death. Further, TG2-R580A significantly potentiated OGD induced cell death, an effect that was negated by targeting it to the nucleus. Treatment of cells expressing wild type TG2, TG2-C277S or TG2-R580A with Compound 4d (Cp4d), a reversible TG2 inhibitor (inhibits transamidation activity) [25] did not cause any protection or potentiation of OGD induced cell death. Intriguingly, treatment of cells expressing either wild type TG2 or TG2-C277S with NC9 (Compound 9 in [26]), an irreversible TG2 inhibitor that reacts with the active site of TG2 and likely stabilizes the protein in an open conformation [26], resulted in a significant increase in OGD-induced cell death. However, NC9 did not cause any protection or potentiation of OGD induced cell death in cells expressing TG2-R580A or in naïve cells. These findings suggest that cellular localization (cytosolic or nuclear) and conformational states are key factors in determining whether TG2 facilitates or ameliorates cell death processes.

## Materials and Methods

### Generation of stable cell lines and cell culture

Naïve, temperature-sensitive immortalized mouse striatal cells were a generous gift from Dr. Marcy E. MacDonald [27]. Cells are maintained in Dulbecco's modified Eagle's medium (DMEM) (Invitrogen, Life Technologies, Inc) with 8% fetal bovine serum (Invitrogen, Life Technologies, Inc.) supplemented with 2 mM L-glutamine (Invitrogen, Life Technologies, Inc.), 100 µg/ml streptomycin (Invitrogen, Life Technologies, Inc.) and 100 units/ml penicillin (Invitrogen, Life Technologies, Inc) at the permissive temperature 33°C. The generation of striatal cells that inducibly express human wild type TG2 was described previously [28]. Striatal cells that inducibly express either TG2-C277S or TG2-R580A were established and characterized as described previously [28]. TG2 expression in these stably transfected cells was induced by incubation with 2 µg/mL doxycycline (Sigma Aldrich, Inc). Naïve cells were also incubated with 2 µg/mL doxycycline as a control.

### *In vitro* transglutaminase assay

*In vitro* transamidation activity was measured in cell extracts using a modification of a previously described procedure [29]. Fifty microliters of assay buffer containing 2 mM CaCl_2_ was used for each reaction. Two hundred fifty microliters of 0.25 M NaOH was added to each tube for termination of reactions. Protein concentration of the supernatant was determined using the bicinchoninic acid assay with bovine serum albumin as a standard, and transamidation activity was calculated after background subtraction as nanomoles of putrescine incorporated per milligram of protein per hour.

### *In situ* transglutaminase assay

*In situ* transglutaminase activity was quantified by determining the incorporation of 5-(biotinamido) pentylamine into protein substrates. We used a microplate assay as described previously [4] with modifications. The cells were labeled for 2 h with 1 mM 5-(biotinamido) pentylamine (Pierce). Forty micrograms of protein was loaded into each well of a 96-well microtiter plate (Falcon-BD Biosciences) in a final volume of 50 µL and the plates were incubated overnight at 4°C. One hundred microliters of HRP-conjugated neutravidin (1∶1000) in 1% BSA and 0.01% Tween 20 in borate saline buffer was used for detecting biotinylated proteins and readings were taken at 492 nm on a microplate spectrophotometer.

### Cell treatment paradigm

Cells were transferred to serum free DMEM media (Invitrogen, Life Technologies, Inc) for the hypoxic treatments or glucose free DMEM media (Invitrogen, Life Technologies, Inc) for OGD. For hypoxic treatments, cells were incubated in a humidified hypoxic glove box (Coy Laboratory Products) containing 5% CO_2_ and 0.1% O_2_ at 33°C for the indicated times (16–36 h). Control cells were incubated in serum free media in a humidified incubator containing 5% CO_2_ and ambient O_2_ at 33°C. Cp4d is a reversible transglutaminase inhibitor [25] and NC9 (Compound 9 in Figure 4 of [26]) is an irreversible transglutaminase inhibitor. Both compounds potently inhibited the *in situ* transamidation activity of TG2, however, NC9 reacts with the active site of TG2 and likely stabilizes an open conformation [26]. For cell death measurements, cells were incubated with or without 20µM Cp4d or 10 µM NC9, which are the highest concentrations that can be used without toxicity, For 36 h in OGD conditions as described above. DMSO was used as vehicle (0.04% v/v) in the treatment media. The control group was treated with DMSO alone.

Cp4d and NC9 were tested individually to determine their ability to inhibit *in situ* transamidation activity in the stable cell lines. For these experiments, cells were incubated in the absence or presence of Cp4d or NC9 and with our without 5µM ionomycin for 3 h prior to *in situ* transamidation activity measures as described above.

### Co-immunoprecipitation

Cells were washed once with ice-cold phosphate buffer saline (PBS) and collected in lysis buffer (10 mM Tris-HCl pH 7.5, 200 mM NaCl, 1 mM EGTA, 1 mM EDTA, 0.75% NP-40 with 0.1 mM phenylmethylsulfonyl fluoride, and 10 µg/ml of each of aprotinin, leupeptin, and pepstatin A). Protein concentrations of the samples were determined by using the bicinchoninic acid assay. Magnetic beads (M-280 Dynabeads, Invitrogen) that were coupled with anti-rabbit IgG were washed 3 times with 2% BSA in PBS. The beads were blocked with 5% ovalbumin in PBS for 2 h at 4°C on an orbital shaker. One microgram of rabbit anti-mouse HIF-1β antibody per sample was added to the beads that were then incubated overnight at 4°C on an orbital shaker. The beads were then washed 3 times with 1% ovalbumin in PBS. One hundred micrograms of each cell lysate were added to the beads that were then incubated for 4 h at 4°C on an orbital shaker. The beads were washed with wash buffer (350 mM NaCl, 0.2% Triton-X in PBS) 4 times. Proteins bound on the beads were collected by boiling for 10 min in 2× stop buffer. The samples were analyzed by western blotting.

### Cellular fractionation

Cells were fractionated as described previously [12] with modifications. Cells were washed once and harvested in ice cold PBS. The cell pellets were resuspended in lysis buffer (10 mM Tris, pH 7.5, 10 mM NaCl, 3 mM MgCl_2_, 0.05% Nonidet P-40, 1 mM EGTA, 0.1 mM phenylmethylsulfonyl fluoride, 10 µg/ml of each of aprotinin, leupeptin, and pepstatin A). Cell lysates were centrifuged at 380×g for 5 min at 4°C. The supernatants were collected as the cytosolic portion and further centrifuged at 100×g for 1 h to clear the cytosolic fractions. The crude nuclei were spun at 1200×g for 10 min at 4°C through a 0.6 M sucrose cushion and the enriched nuclei were collected in the pellet. The proteins were visualized by immunoblotting.

### Immunoblotting

Cell lysates were blotted as described [12] with modifications. The blots were probed overnight at 4°C with anti-TG2 antibody TG100 (Neomarkers) (1∶2000) or anti-HIF-1α antibody (Calbiochem Inc) (1∶1000), anti-HIF-1β (Novus Biologicals) (1∶2000), anti-histone (Chemicon Inc.) (1∶10.000), anti-α tubulin (Sigma-Aldrich Inc.) (1∶10.000) or anti-actin (Chemicon Inc.) (1∶5000) antibodies in 5% non-fat dry milk in TBST. The membranes were rinsed three times for 30 min with TBST and incubated with HRP-conjugated secondary antibody (1∶2000) in 5% non-fat dry milk in TBST for 2 h at room temperature. Lastly, the blots were rinsed three times for 1 h with TBST and developed with a chemiluminescence solution as previously described [30].

### HRE-Luciferase reporter assay

Cells were transiently transfected with firefly luciferase vector pGL3-SV40–6HRE, a generous gift from Dr. Carine Michiels. Co-transfection with renilla luciferase vector was carried out using Lipofectamine 2000 reagent according to manufacturer's directions. After 24 h, cells were transferred to serum free media and incubated under hypoxic conditions for 16 h. Control cells were kept in serum free media under ambient O_2_ conditions. Luciferase activity was measured by using the Dual-Luciferase Reporter Assay System Kit (Promega) according to the manufacturer's protocol.

### Lactate Dehydrogenase (LDH) Release Assay

Cells were transferred to glucose free media and incubated in a humidified hypoxic glove box at 0.1% O_2_, 5% CO_2_ at 33°C for 36 h. Control samples were kept in serum free media in a humidified chamber at 5% CO_2_, 33°C and ambient O_2_ level. LDH release was measured by using an LDH release assay kit (Roche) according to the manufacturer's protocol.

### Calcein AM cell viability assay

Cells were transferred to glucose free media and incubated in a humidified hypoxic glove box at 0.1% O_2_, 5% CO_2_ at 33°C for 24 h. At the end of the treatment, cells were washed once with phosphate buffer saline (PBS) and Calcein AM in PBS was added to the wells to a final concentration of 5 µM. The cells were kept at 33°C for 30 min and fluorescence readings were taken using a 490 nm excitation filter and a 520 emission filter (BioTek Synergy HT Multi-Detection Microplate Reader). The fluorescence intensity is proportional to the number of viable cells.

### Lentivirus production and transduction

Wild type TG2, TG2-R580A and the nuclear localization signal tagged TG2-R580A (NLS-R580A) were cloned into FIGB lentiviral vector. TG2 expression was driven by a CMV promoter, with GFP downstream under the control of IRES [31]. The lentiviral vector backbone was a generous gift from Dr. C. Proschel at the University of Rochester. Viral particles were made in HEK 293T cells (ATCC, cat. no. CRL-11268) by cotransfection of the lentiviral vector, pMD2.G (VSVG-envelope) and psPAX2 (packaging components) plasmids (Addgene.org). Viral particles were concentrated 20 times by centrifugation of cell media at >50,000×g for 2 h at 4°C. The pellet was resuspended in 1% BSA in sterile PBS.

Cells were plated at 75% confluency on 24-well plates in low serum media (DMEM with 2% FBS) and after 24 h viral particles (30–35 µl) were added directly to the media of the cells. Cells were incubated overnight in the humidified incubator at 5% CO_2_ at 37°C, the restrictive temperature that prevents proliferation, and kept for 5–6 days to allow for expression of the transduced constructs. The media was half-replaced with fresh media every 3 days. Expressions of constructs were confirmed by immunocytochemistry and western blot analysis.

### Immunocytochemistry

Naïve cells were plated at 75% confluency on coverslips in low serum media and transduced as described above. After 6–7 days of infection, cells were fixed with 4% paraformaldehyde for 15 min, washed once with PBS and kept in 0.1 M glycine for 5 min. The cells were permeabilized with 0.2% Triton-X in PBS and blocked with 2% BSA in PBS for 2 h at room temperature. Cells were incubated with anti-human TG2 antibody TG100 (1∶500 dilution in PBS) overnight. The next day, cells were washed twice with PBS and incubated with FITC-conjugated secondary antibody for 2 h at room temperature. The cells were washed twice with PBS and incubated with Hoechst nuclear stain (1∶2000) for 15 min at room temperature. After rinsing twice with PBS, the coverslips were mounted on glass and viewed using an Axiovert inverted microscope with Axiocam-XMR camera (Carl Zeiss, Hamamatsu ORCA-ER digital camera). Hoechst staining was used to determine the total number of cells. The FITC positive cells represent lentiviral expression of TG2. The merged images of FITC and Hoechst staining were used to determine the percent of cells that were transduced.

### Statistics

All data were expressed as mean +/− SEM and were plotted using Graphpad Instat software. The means were compared with ANOVA and Tukey's test in all the figures except the in situ transamidation activity experiment performed with Cp4d in which unpaired t-test was used.

## Results

### Stable expression of TG2 constructs

**Figure 1:**
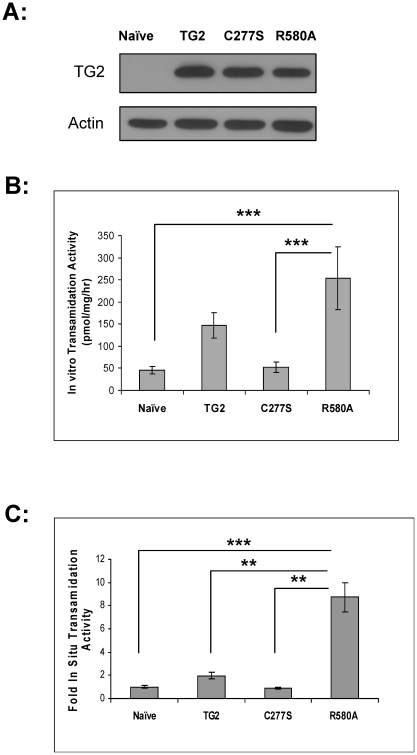
Characterization of striatal stable cell lines. **A**, Representative immunoblots of cell lysates from striatal cells stably expressing wild type TG2, TG2-C277S (C277S) or TG2-R580A (R580A) mutants. Top panel: Lysates were blotted for TG2. Bottom panel: Lysates were blotted for actin as loading control. **B**, *In vitro* transamidating activities of stable striatal cell lines. TG2-R580A cells show significantly greater *in vitro* transamidating activity compared to naïve and C277S cells. (N = 5) **C**, *In situ* transamidating activity of striatal stable cell lines. TG2-R580A (R580A) cells show significantly higher *in situ* transamidating activity than all the other cell lines. (N = 3) Results are shown as mean +/− standard error, **p<0.01, ***p<0.005.

To determine the relative expression of the TG2 constructs in the stably transfected cells, lysates were immunoblotted with anti-human TG2 antibody which only recognizes exogenous TG2 after induction by doxycycline treatment. The blots in Fig. 1A show that the stably transfected cells express TG2, TG2-C277S and TG2-R580A at equivalent levels. The apparent molecular weight of TG2 was ∼77 kDa. Approximately 30–40% of the total number of cells express detectable levels of the transgene in the stable cell lines (data not shown). Immunoblotting revealed that the TG2 transgene expression in the stable cell lines was substantially higher than the levels of endogenous TG2 in mouse or human brain lysates (data not shown). However, the brain lysates contains many cell types other than neurons and the expression level of TG2 in the different cell types is highly variable. Therefore it is difficult to compare expression levels in the cell lines and in neuronal populations in vivo.

### *In vitro* transglutaminase activity of stable cell lysates

To determine the total transamidation activity of the stably transfected cell lines, an *in vitro* transglutaminase assay was used. Cell lysates were collected from each cell line and activity was measured in the presence of 2 mM calcium. This concentration of calcium was determined to give maximum *in vitro* transamidating activity in these cell lines (data not shown). The *in vitro* transamidating activities of each cell line are shown as pmol/mg/h (Fig. 1B). TG2 overexpressing cells showed 3-fold higher *in vitro* transamidating activity compared to naïve cells. TG2-C277S (a transamidating-inactive form) overexpressing cells showed approximately equal transamidating activity as the naïve control cells. TG2-R580A overexpressing cells showed significantly higher (5-fold) *in vitro* transamidating activity compared to naïve control. These results indicate that all the stable cell lines show the expected *in vitro* transamidating activities.

### *In situ* transglutaminase activity of stable cells

To determine intracellular transamidating activities of the stably transfected cell lines, an *in situ* transglutaminase assay was used. Cells were labeled for 2 h with 1 mM 5-(biotinamido) pentylamine and incorporation of this substrate to the intracellular proteins was measured under basal conditions (Fig. 1C). TG2 overexpressing cells showed a 2 fold increase in intracellular transamidating activity. There was no increase in the intracellular transamidating activity of TG2-C277S overexpressing cells due to the lack of catalytic activity. The TG2-R580A cells showed 8-fold higher *in situ* transamidating activity compared to naïve control cells which was expected due to the more open conformation of the protein and the lack of inhibition by intracellular GTP [9], [23].

### The interaction of TG2 with HIF-1β

**Figure 2:**
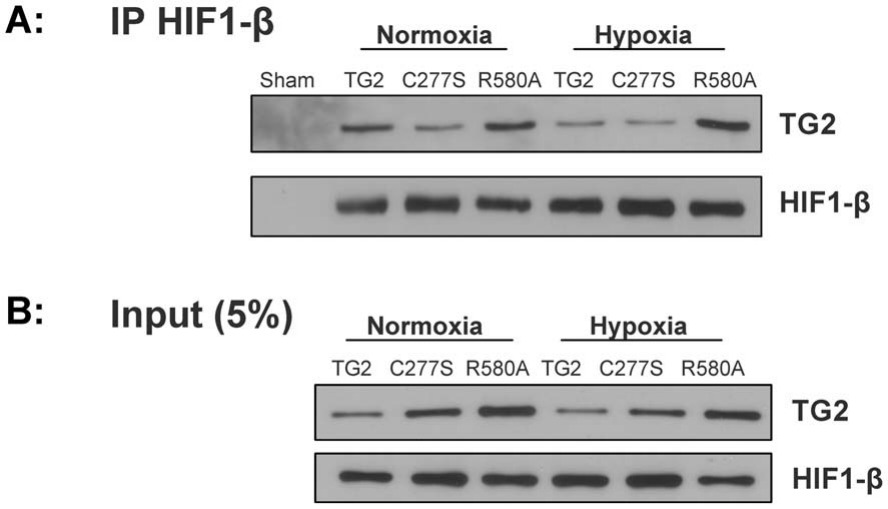
Interaction of TG2 with HIF-1β. **A**, Representative immunoblots showing the coimmunoprecipitation of TG2 with HIF-1β. Top panel: HIF-1β was immunoprecipitated from the cell lysates of stable striatal cell lines and blotted for TG2. Bottom panel: The top blot was stripped and re-probed for HIF-1β. Wild type TG2, and the mutants TG2-C277S (C277S), TG2-R580A (R580A) immunoprecipitated with HIF-1β. **B**, 5% of the total amount of cell lysate used for immunoprecipitation was blotted for TG2 and HIF-1β.

The interaction of TG2 with HIF-1β was described previously [2]. To further characterize the striatal cell lines and to test whether TG2-R580A can interact with HIF-1β, co-immunoprecipitation experiments were performed. HIF-1β was pulled down from cell lysates and the precipitates were blotted for TG2 (Fig. 2A top panel). TG2, TG2-C277S, and TG2-R580A were detectable in the immunoprecipitated cell lysates under both normoxic and hypoxic conditions. There was no detectable TG2 in the negative control sample (sham) in which the immunoprecipitation was performed with magnetic beads but without the HIF-1β antibody. The immunoprecipitation blot was reprobed with HIF-1β antibody to show the presence of HIF-1β in the immunoprecipitates (Fig. 2A bottom panel). Five percent of the total protein amount used for immunoprecipitation, was blotted for TG2 (Fig. 2B top panel) and HIF-1β (Fig. 2B bottom panel) as input controls. These data suggest that the interaction of TG2 with HIF-1β is independent of its transamidation activity or GTP binding ability since both TG2-C277S (transamidating inactive) and TG2-R580A (GTP binding deficient) mutants interact with HIF-1β.

### Intracellular localization of TG2, TG2-C277S and TG2-R580A in stable cells

**Figure 3:**
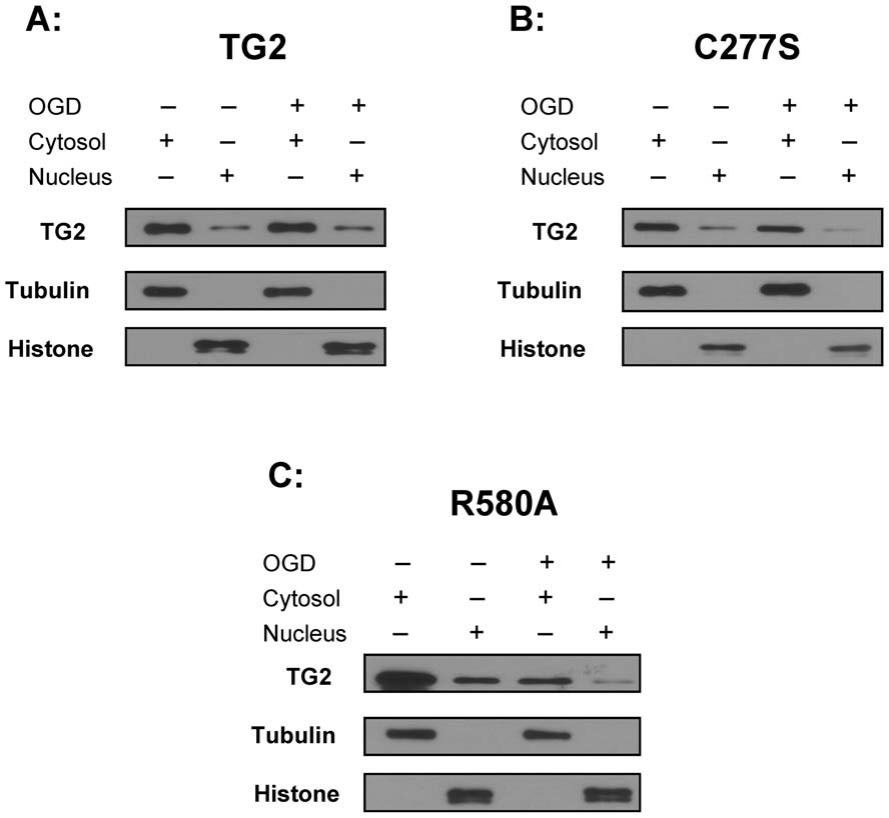
Subcellular localization of TG2, TG2-C277S and TG2-R580A. **A**, **B**, **C**, Immunoblots showing subcellular localization of TG2, TG2-C277S (C277S) and TG2-R580A (R580A). Striatal stable cells were fractionated into nuclear and cytosolic compartments with or without OGD treatment. Cell lysates were blotted for TG2 (1^st^ panels), histones (2^nd^ panels), tubulin (3^rd^ panels). Histone and tubulin blots show the purity of the fractions. TG2 blots show that there is no increase in the nuclear TG2 levels after OGD treatment in any of the cell lines.

It has been shown that nuclear TG2 levels increase in SH-SY5Y cells in response to hypoxic treatment [2] and TG2 translocates to the nucleus in mouse brain after MCAL [20]. To determine if TG2, TG2-C277S or TG2-R580A localize to the nucleus in this cell line in response to OGD, the cells were fractionated into cytosolic and nuclear portions and immunoblotted for TG2 (Fig. 3A–C, top panels). Fractions were also blotted with total histone (nuclear protein) and α-tubulin (cytosolic protein) antibodies to determine the purity of the fractions. As expected, histones were only detectable in the nuclear fractions and α-tubulin was only present in cytosolic fractions. Nuclear levels of wild type TG2 did not change after OGD treatment in TG2-expressing cells (Fig. 3A). However, there were decreases in the nuclear levels of TG2-C277S (Fig. 3B, ∼70% less) and TG2-R580A (Fig. 3C, ∼66% less) as well as the cytosolic levels of TG2-R580A after OGD treatment. These data indicate that in striatal cells TG2 localization to the nucleus does not increase in response to OGD.

### HRE reporter activity in response to hypoxia

**Figure 4:**
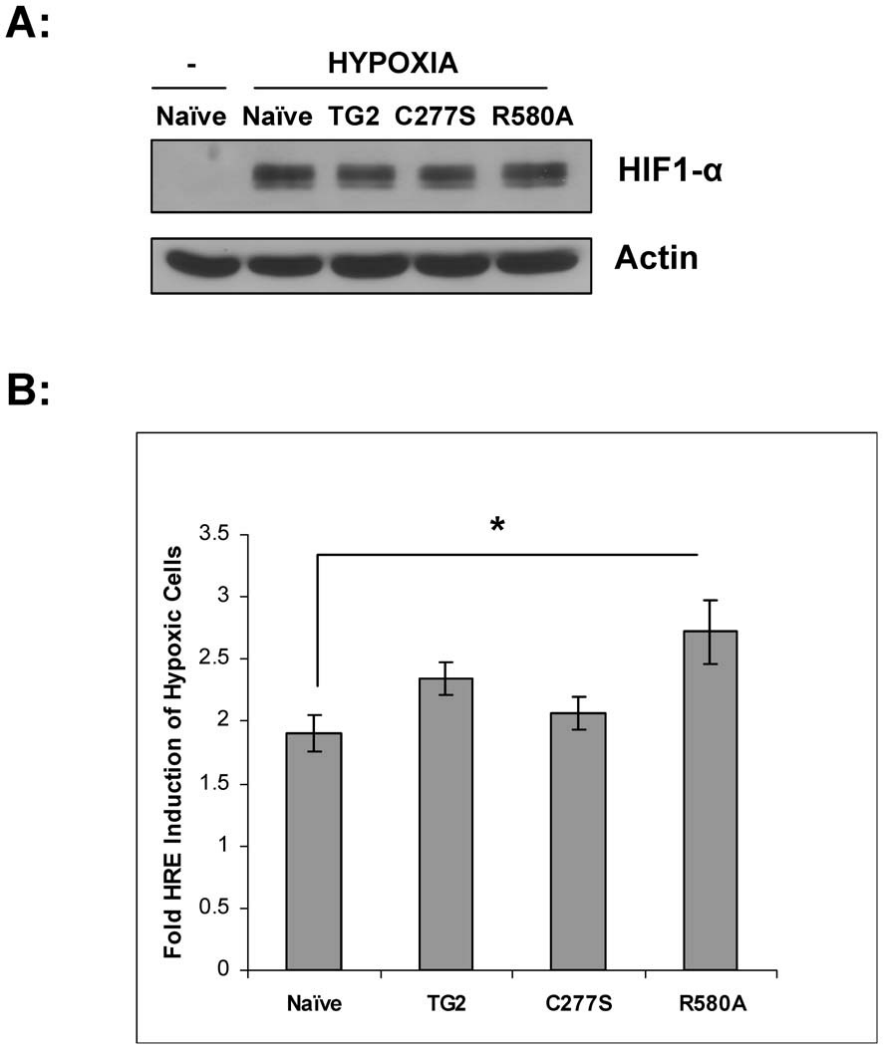
HRE luciferase assay. **A**, Immunoblots of the cell lysates from striatal stable cells showing the HIF-1α protein stabilization after 16 h of 0.1% O_2_. (−) represents naïve cells maintained in normoxic conditions. Lysates were also probed for actin as a loading control. **B**, Fold HRE luciferase activity of stable striatal cells after 16 h of 0.1% O_2_. TG2-R580A (R580A) cells showed significantly higher HRE activation than the other cell lines. (N = 8) Results are shown as mean +/− SE, *p<0.05.

Cells were transferred to serum free media and maintained at 0.1% O_2_ in a humidified hypoxic chamber prior to collecting lysates and blotting with a HIF-1α antibody (Fig. 4A top panel). HIF-1α protein was not detectable under normoxic conditions, as expected, and the levels of HIF-1α were equivalent in all the cell lines after hypoxic treatment. An actin blot is shown as a loading control. The effects of TG2, TG2-C277S and TG2-R580A on HRE reporter activity were also measured (Fig. 4B). There was no significant difference in HRE luciferase activity of TG2 and TG2-C277S overexpressing cells compared to naïve control cells. However, cells overexpressing TG2-R580A showed significantly higher HRE reporter activity than naïve control cells. This experiment was repeated using at least one other independent subclone of each cell line, and the results were approximately the same (data not shown).

### TG2-R580A potentiates OGD induced cell death

**Figure 5:**
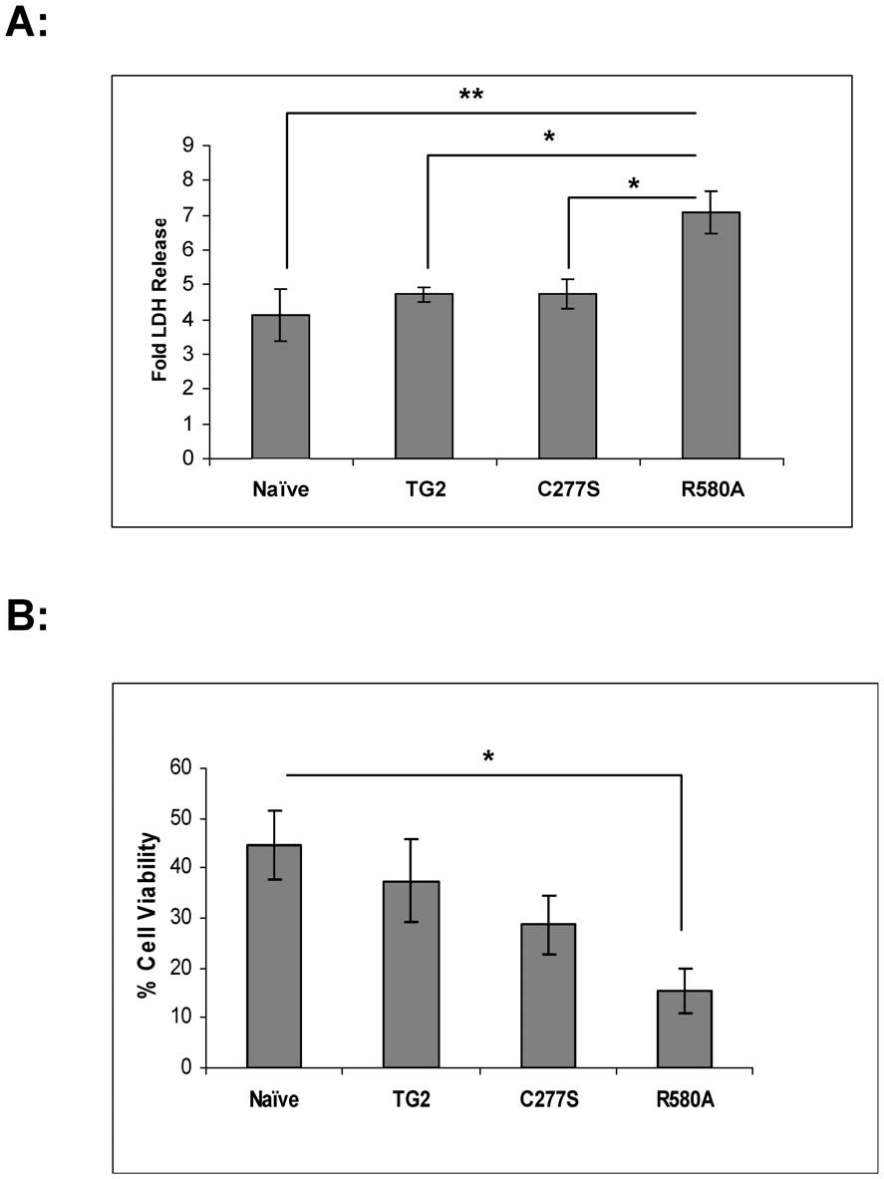
OGD-induced cell death. **A**, Fold LDH release after 36 h of OGD at 0.1% O_2_. TG2-R580A (R580A) cells showed significantly higher fold LDH release than other cell lines after OGD treatment. (N = 6) **B**, Calcein AM cell viability assay after 24 h OGD treatment at 0.1% O_2_. TG2-R580A (R580A) cells showed significantly lower cell viability after OGD treatment. (N = 6) Results are shown as mean +/− SE, *p<0.05, ** p<0.01.

To test the effect of TG2-R580A in OGD-induced cell death, LDH release assay was performed subsequent to 36 h of OGD. Naïve, TG2 and TG2-C277S cells showed 4-fold and TG2-R580A cells showed 7-fold increases in LDH release after OGD treatment compared to their relative normoxic controls. Interestingly, TG2-R580A cells showed significantly greater fold LDH release (1.5- to 1.7-fold) than naïve, TG2 and TG2-C277S cells after OGD treatment (Fig. 5A). These data suggest that TG2-R580A expression facilitates OGD-induced cell death in these cells. This experiment was repeated using another independent subclone of each cell line, and the result was approximately the same. We also carried out the same experiment in the absence of Doxycyclin, which induces exogenous TG2 expression. When we did not induce TG2 expression OGD-induced cell death in the individual stable cell lines and the naïve cells no differences in the extent of cell death were observed (data not shown).

To further examine the effects of the different TG2 constructs on OGD-induced cell death, a viability assay with Calcein AM was carried out. This assay measures the intracellular esterase activity present in viable cells and thus the readings represent the amount of viable cells, which is normalized to the normoxic conditions. After 24 h of OGD treatment, naïve cells showed 45% cell viability. The viability of TG2-R580A cells was significantly reduced to 15% after OGD treatment (Fig. 5B).

### Targeting TG2-R580A to the nucleus counteracts the detrimental effect of TG2-R580A in OGD

**Figure 6:**
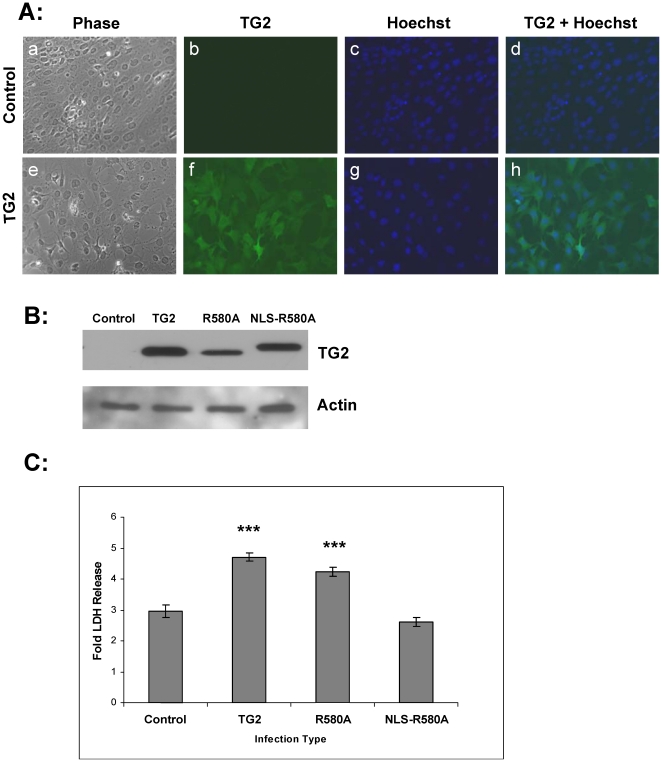
OGD-induced cell death with NLS tagged R580A. **A**, Immunocytochemistry of naïve striatal neurons that were infected with a control or wild type TG2 lentiviral construct. Panels a, b, c, and d represent the naïve cell transduced with control lentivirus. Panels e, f, g, and h represent naïve cells transduced with wild type TG2. Immunocytochemistry was performed with a TG2 antibody that was recognized by a FITC-conjugated mouse secondary antibody. From left to right: phase, TG2 staining labeled by FITC, Hoechst nuclear staining, merge of FITC and Hoechst staining. **B**, Immunoblots of cell lysates from naïve cells transduced with control, TG2, TG2-R580A (R580A) and NLS tagged R580A (NLS-R580A) lentiviruses. Actin blot is shown for loading controls. **C**, Fold LDH release in lentivirus infected striatal cells after 36 hr of OGD at 0.1% O_2_. Both TG2 and TG2-R580A (R580A) expressing cells showed significant increases in fold LDH release after OGD treatment, while NLS-R580A showed similar fold LDH release amount as the control. (N = 8) Results are shown as mean +/− SEM, ***p<0.001.

To test whether nuclear localization of TG2-R580A alters its detrimental effects, naïve cells were transduced with lentiviral constructs that express wild type TG2, TG2-R580A or NLS-R580A. The cells were transduced with control virus (Fig 6A: a, b, c, d) and TG2-expressing virus (Fig 6A: e, f, g, h) and after 5–6 days the cells were fixed and probed with a TG2 antibody that recognizes only exogenously expressed human TG2. The merged image (Fig. 6A: h) shows that >95% of cells are transduced with the TG2 virus. In order to compare the expression of TG2, TG2-R580A and NLS-R580A, transduced cell lysates were blotted with the TG2 antibody (Fig. 6B, top panel). An actin blot is shown as loading control (Fig. 6B, bottom panel). All TG2 constructs were expressed at comparable levels. The nuclear localization of NLS-R580A was confirmed by previous studies performed in our lab [5].

Transduced cells were treated with OGD and cell death was measured. Both TG2 (60%) and TG2-R580A (44%) transduced cells showed significant induction of LDH release compared to control (Fig 6C). However, cell death in the NLS-R580A transduced cells was similar to that observed in control cells indicating that localization of TG2 to the nucleus prevents it from acting in a pro-death capacity.

### Cp4d (a reversible TG2 transamidation inhibitor) does not prevent the detrimental effect of TG2-R580A in ischemic cell death

**Figure 7:**
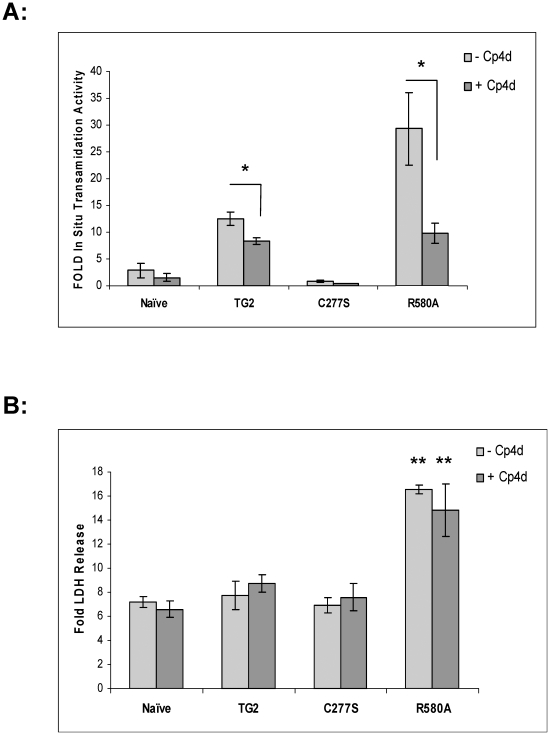
OGD-induced cell death with Cp4d Treatment. **A**, Fold increase in *in situ* transamidation activity in response to ionomycin treatment in the absence (−) or presence (+) of 20µM Cp4d (Reversible TG2 inhibitor) in comparison to basal *in situ* transamidation activity of the corresponding cell line. TG2-R580A cells showed potentiation of *in situ* transamidation activity with 5µM ionomycin treatment which was inhibited by Cp4d treatment (N = 3) **B**, Fold LDH release after 36 h of OGD treatment at 0.1% O_2_ in the absence (−) or presence (+) of 20 µM Cp4d (Reversible TG2 inhibitor) in comparison to percent LDH release amount of corresponding cell line at normoxia. None of the cell lines showed significant changes in fold LDH release with Cp4d treatment. (N = 3) Results are shown as mean +/− SE, * p<0.05. **p<0.01.

To test whether the high basal transamidation activity of TG2-R580A is necessary for its ability to potentiate cell death, stably transfected cells were treated with OGD with or without 20µM Cp4d. This concentration was the highest non-toxic concentration of Cp4d that we could use. To demonstrate that Cp4d inhibits *in situ* transamidation activity of TG2 and TG2-R580A, cells were incubated with or without 5µM ionomycin in the absence or presence of 20µM Cp4d (Fig. 7A). Ionomycin increases intracellular Ca^2+^ levels and resulted in a significant increase in intracellular transamidation activity of TG2 and TG2-R580A. *In situ* transamidation activity is expressed as fold increase relative to the corresponding stable cell line without ionomycin treatment (Fig. 7A). Cp4d significantly attenuated the ionomycin-induced increase in the transamidation activity of both TG2 and TG2-R580A. We also confirmed by immunocytochemistry that TG2 is not externalized in this cell model (data not shown). Therefore we can conclude that Cp4d inhibits the elevation of *in situ* transamidation activity in response to ionomycin by acting intracellularly. LDH release was measured to detect cell death in stable cell lines in the absence and presence of 20 µM Cp4d. Fold LDH release was expressed relative to the corresponding stable cell line maintained under normoxic conditions (Fig. 7B). Naïve, TG2 and TG2-C277S cells showed a 6–8 fold increase, while TG2-R580A cells showed a 16 fold increase in LDH release in response to OGD treatment. Cp4d treatment did not result in any changes in LDH release in TG2, TG2-C277S and TG2-R580A cells after OGD treatment. Cp4d also did not have any affect on basal LDH release in normoxic conditions. Given that inhibition of the transamidating activity of TG2-R580A did not attenuate its effect on OGD-induced cell death, it is likely that the potentiation of cell death by TG2-R580A cells in response to OGD treatment is transamidation independent.

**Figure 8:**
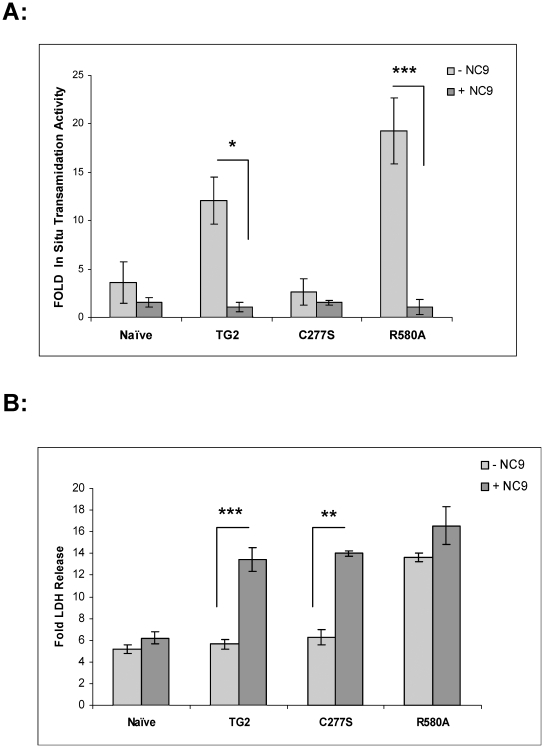
OGD-induced cell death with NC9 treatment. **A**, Fold *in situ* transamidation activity with ionomycin treatment in the absence (−) or presence (+) of 10 µM NC9 (Irreversible TG2 inhibitor) in comparison to basal *in situ* transamidation activity of the corresponding cell line. TG2 cells (12 fold) and TG2-R580A cells (19 fold) showed potentiation of *in situ* transamidation activity with 5µM ionomycin treatment which was inhibited 100% by NC9 treatment (N = 3). **B**, Fold LDH release after 36 h of OGD treatment at 0.1% O_2_ in the absence (−) or presence (+) of 10 µM NC9 (TG2 inhibitor) in comparison to percent LDH release amount of corresponding cell line at normoxia. TG2 and TG2-C277S (C277S) cells showed significant increases in fold LDH release with NC9 treatment. (N = 4) Results are shown as mean +/− SE, *p<0.05, **p<0.01, ***p<0.001.

We also tested an irreversible transglutaminase inhibitor NC9 which reacts with the active site of TG2 [26] and likely stabilizes the protein in open conformation [7]. NC9 significantly inhibits the *in situ* transamidation activity of TG2 and TG2-R580A (Fig. 8A). NC9 was used at the highest non-toxic concentration and at this concentration it was fully inhibitory. LDH release was measured and expressed as fold increase relative to the corresponding stable cell line maintained at normoxic conditions (Fig. 8B). Naïve, TG2 and TG2-C277S cells showed ∼4- to 5-fold increase, and TG2-R580A expressing cells showed ∼12-fold increase in LDH release after OGD treatment. NC9, which inhibits the transamidation activity of TG2, did not have any affect on basal LDH release in stable cells in control normoxic conditions. Interestingly, both TG2 (2.65-fold) and TG2-C277S (2.5-fold) expressing cells showed a significant increase in OGD-induced LDH release in response to NC9 treatment. However, NC9 treatment did not have any effect on OGD-induced LDH release in naïve cells or cells expressing TG2-R580A (Figure 8B).

## Discussion

In this study, we investigated how the transamidation activity state, cellular localization and conformation of TG2 impact its effect on OGD induced cell death. We used TG2-C277S as a transamidation inactive form and TG2-R580A as a GTP-binding deficient form having greater transamidating activity. We also used Cp4d, a reversible transglutaminase inhibitor [25] to determine the role of transamidation activity of TG2 in how it regulates OGD induced cell death.

*In vitro* and *in situ* transamidation activities of the stable cell lines were measured and confirmed to be as predicted according to previous studies [8], [9]. We also demonstrated that, as previously observed in SH-SY5Y cells [2], wild-type TG2 and TG2-C277S co-immunoprecipitated with HIF-1β. We extended these findings by demonstrating that TG2-R580A interacts with HIF-1β to approximately the same extent as wild type TG2 and TG2-C277S. Therefore, TG2 interacts with HIF-1β independent of its transamidation activity state or GTP binding ability.

In SH-SY5Y cells and cortical neurons TG2 translocates to the nucleus and attenuates HRE reporter activity under hypoxic conditions [2]. In addition, in a mouse stroke model in which human TG2 was expressed in neurons, translocation of TG2 into the nucleus was observed [20]. In contrast, TG2 did not localize to the nucleus in these clonal striatal cells in response to hypoxia and we did not observe a suppression effect on HRE reporter activity. In fact, decreases in the nuclear levels of TG2-C277S and TG2-R580A, as well as total TG2-R580A protein level were observed after OGD treatment. Binding of GTP is required for TG2 to adopt the most compact and protease resistant conformation [3], [9], [32] and therefore TG2-R580A may be more susceptible to proteolysis which may be a contributing factor to the OGD-induced decreases in TG2-R580A levels. Expression of TG2-R580A resulted in a slight but significant increase in HRE activity compared to naïve control cells while TG2 and TG2-C277S expressing cells did not show any difference. These findings indicate that the interaction of TG2 with HIF1β is not sufficient to mediate its ability to suppress HRE activity or facilitate its translocation to the nucleus in response to hypoxia. Further, the hypoxic-induced translocation of TG2 to the nucleus is cell type specific and thus likely to be dependent on other proteins or factors. The mechanism of nuclear translocation of TG2 is still unknown. It has been suggested that TG2 has two NLS sequences [17], therefore can interact with importin α-3 and may be localized to nucleus via this interaction [33]. Finally, these findings support the hypothesis that the suppression of HRE activation by TG2 is dependent on its nuclear localization.

TG2 can both ameliorate and facilitate cell death processes in a cell type and stress dependent manner [34], [35], and it has been previously suggested that transamidating activity and subcellular localization are contributing factors in determining how TG2 affects cell viability. When the stressor increased the transamidating activity of TG2 in SH-SY5Y cells, TG2 facilitated apoptosis. In contrast, if the stressor did not result in increased transamidating activity, TG2 ameliorated cell death [29]. In HEK cells, nuclear-targeted TG2-C277S protected against thapsigargin-induced cell death [12]. In this study, TG2-R580A expression significantly increased OGD-induced cell death compared to the expression of TG2 or TG2-C277S, or naïve cells. However targeting TG2-R580A to the nucleus (NLS-R580A) abrogated this response. Previously, in HEK cells, TG2-R580A increased cell death in response to thapsigargin treatment while nuclear targeted TG2-R580A did not show the same detrimental effect [5]. These findings strongly suggest that nuclear localization of TG2 is protective and results in increased cell survival. Given the fact that TG2-R580A exhibits higher basal transamidating activity and adopts a less compact conformation, [9], [23] we next investigated what role each of these variables play in determining the effects of TG2-R580A on OGD-induced cell death.

In order to determine whether transamidation activity is critical for the detrimental role of TG2-R580A, we used the reversible TG2 transamidation inhibitor Cp4d [25] which significantly inhibited *in situ* transamidation activity of TG2 and TG2-R580A cells. However, Cp4d treatment did not result in any potentiation or suppression of cell death in any of the stable cell lines in response to OGD. Therefore, we can conclude that the cell death affect of TG2-R580A is transamidation independent.

We also tested an irreversible TG2 transamidating inhibitor NC9 [26]. NC9 bears an electrophilic group designed to react with the active site thiol and strongly inhibited the *in situ* transamidation activity of TG2-R580A in stable cells. NC9 binds to the active site of TG2 irreversibly [26] and therefore likely stabilizes the protein in an open, but inactive conformation [7]. Intriguingly, treatment of cells expressing TG2 or TG2-C277S with inhibitor NC9 resulted in a significant increase in OGD-induced cell death. However, NC9 had no effect on OGD-induced cell death in naïve cells or TG2-R580A expressing cells. Given that NC9 increased OGD-induced cell death in the presence of TG2-C277S (a transamidation inactive mutant), it is possible that NC9 is electrophilic enough to react with the nucleophilic Ser residue in the active site of TG2-C277S [26] which would result in a more extended conformation as well. On the other hand, TG2-R580A, which already showed significantly increased OGD-induced cell death, exhibited neither an attenuation nor potentiation of cell death in response to NC9. It has been suggested that TG2-R580A is already in a more open conformation [9], and therefore it can be speculated that although NC9 reacts with the active site of TG2-R580A and inhibits its transamidating activity, it does not significantly extend the conformation of the molecule. Therefore the potentiation of OGD-induced cell death in TG2-R580A cells is not dependent on high transamidation activity which can be inhibited by both irreversible and reversible inhibitors.

Overall, these data suggest that the pro-death role of TG2-R580A is not due to its high basal transamidation activity, but it is possible that the more open conformation of TG2 in the cytosol results in facilitation of cell death processes. Given the dramatically different conformations in the GTP-bound state (closed and compact) and the activated state (open and extended) [7], [8], [9], it can be suggested that they may play an important role in regulating TG2's interaction with other proteins, and that these non-enzymatic interactions may be critical in determining the function of TG2, either facilitating or ameliorating cell death processes.

Interestingly Protein 4.2, which is the only catalytically inactive member of transglutaminase family, is primarily a linker protein interacting with several membrane proteins (B3, ankyrin, spectrin, CD47) in erythrocytes [36], [37], [38]. Based on TG2 structural studies, it has been suggested that Protein 4.2 might require an open conformation to interact with other protein partners like B3 (Band 3) on the plasma membrane [39]. Given the similarities between TG2 and Protein 4.2, it is tantalizing to speculate that the primary function of TG2 in cell death/survival processes is to act as a scaffold or linker protein rather than as an enzyme and that the conformational changes it undergoes dictate its binding partners and thus different functions.

In conclusion, these results indicate that when TG2 is in the cytosol in a more extended conformation it facilitates OGD-induced cell death independent of its transamidation activity. Additionally, hypoxic-induced relocalization of TG2 to the nucleus is cell type specific and plays primarily a protective role. Overall these findings provide important new insights into the differential effects of TG2 on cell survival and cell death processes.
